# Impact of Aerobic Exercise on Oxygenation, Pulmonary Function, and Nasal Nitric Oxide in Primary Ciliary Dyskinesia

**DOI:** 10.2174/0118743064365386250212050147

**Published:** 2025-02-18

**Authors:** Gabriel Gonzalez-Diaz, Zachary J. Demetriou, Jose Muñiz-Hernandez, Marcos J. Ramos-Benitez, Ricardo A. Mosquera, Wilfredo De Jesús-Rojas

**Affiliations:** 1 Ponce Health Sciences University, School of Medicine, Ponce, PR, 00716, United States; 2 San Juan Bautista, School of Medicine, Caguas, PR, 00727, United States; 3 McGovern Medical School, University of Texas Health Science Center at Houston, Houston, TX 77030, United States

**Keywords:** Primary Ciliary Dyskinesia, Exercise, Oxygen Saturation, Nitric Oxide, Respiratory Physiology, Quality of Life, *RSPH4A*

## Abstract

**Background:**

Primary Ciliary Dyskinesia (PCD) is a rare genetic disorder characterized by impaired mucociliary clearance, resulting in chronic respiratory complications. While exercise benefits respiratory health, its impacts on PCD remain understudied.

**Objective:**

The objective of this study was to assess how moderate aerobic exercise influences FEV1, SpO2, and nNO levels in PCD patients, with a focus on short-term post-exercise changes.

**Methods:**

This is a matched case-control pilot study involving 12 PCD patients homozygous for the RSPH4A (c.921+3_921+6del) mutation and 12 healthy controls (HC). Baseline FEV1, SpO2, and nNO levels were measured before participants underwent a six-minute exercise challenge test (ECT) on a stationary bicycle. Post-exercise measurements included FEV1 at 5, 10, 15, and 20 minutes, nNO after final spirometry, and SpO2 at 5 minutes.

**Results:**

The PCD group experienced a significant increase in SpO2 from 95.5% ± 2.1 to 97.7% ± 1.5 post-exercise (p < 0.05), while the HC group had stable SpO2 levels with a minor increase from 97.9% ± 1.5 to 98.9% ± 1.4 (p = 0.14). No significant changes in FEV1 or nNO levels were observed post-exercise in either group. One HC participant exhibited exercise-induced bronchoconstriction.

**Conclusion:**

Aerobic exercise improves oxygenation in PCD patients without adverse effects on pulmonary function or nNO levels. Further research is necessary due to the small sample size and genetic homogeneity to confirm these findings and evaluate long-term outcomes. Moreover, this pilot study highlights the safety and potential respiratory benefits of aerobic exercise in PCD patients, supporting further investigation into its role in clinical management.

## 1 INTRODUCTION

Primary Ciliary Dyskinesia (PCD) is a rare autosomal recessive genetic disorder characterized by defective motile cilia, leading to chronic respiratory tract infections, reduced nasal Nitric Oxide (nNO) levels, and various other complications [1, 2]. The disease has a prevalence ranging from approximately 1 in 10,000 to 40,000 individuals [3]. Among Hispanic populations, such as Puerto Rico, the prevalence is estimated at 1 in every 16,309 individuals [4]. In Puerto Rico, a founder mutation in the *RSPH4A* gene (c.921+3_921+6del) has been identified in Puerto Rican patients, with PCD significantly impacting the diagnostic landscape and patient management on the island [5-7].

nNO, a biomarker typically reduced in PCD, plays a crucial role in diagnosing the disease [8]. Studies show significantly lower nNO levels in patients with PCD compared to healthy controls, a finding that aids in differential diagnosis [9]. The decreased levels have been well established by multiple studies, and a cutoff of 77 nL/min has been widely accepted since 2013 [10]. Although the physiological mechanisms underlying nNO production in PCD are not well understood, it is thought to be partly due to defects or regulation of Nitric Oxide Synthase (NOS) in the respiratory epithelium [11].

PCD management typically includes airway clearance techniques, antibiotics, and supportive therapies, with exercise being a potentially beneficial yet underexplored intervention [12]. Exercise is known to improve pulmonary function and overall health in other chronic respiratory conditions, such as cystic fibrosis and asthma, highlighting its potential relevance for PCD [13, 14]. Despite these potential benefits, the physiological response to exercise in these patients remains understudied, particularly regarding its effects on lung function parameters like Forced Expiratory Volume in one second (FEV1) and oxygen saturation (SpO2). These parameters are critical measures of pulmonary function often found to be below normal levels in PCD [9]. Understanding how these parameters respond to exercise is essential for developing safe and effective physical activity guidelines for this population.

This study aims to address these gaps by evaluating the immediate effects of aerobic exercise on FEV1, nNO levels, and SpO2 in patients with PCD, particularly focusing on those with the *RSPH4A* (c.921+3_921+6del) genetic variant. Given the limited research in this area, our study sheds light on valuable insights that could inform clinical guidelines and improve the quality of life for patients with PCD through tailored exercise regimens. By characterizing the exercise-induced changes in key physiological parameters, we hope to establish a foundation for further research and clinical practice improvements in PCD.

## 2 MATERIALS AND METHODS

### 2.1 Study Design

A matched case-control pilot study was conducted to evaluate the immediate effects of aerobic exercise on pulmonary function, nNO levels, and SpO2 in patients with PCD to healthy controls (HC). The study was completed at the Puerto Rico PCD Center, which is accredited by the PCD Foundation. To minimize selection bias, participants were consecutively recruited from the PCD Center, ensuring a representative sample of the population under study. The design included detailed pre- and post-exercise assessments to identify specific exercise-induced changes unique to the PCD population.

A sample size of 12 patients (*n*=12) with PCD homozygous for the *RSPH4A* (c.921+3_921+6delAAGT) genetic variant was selected based on disease prevalence in Puerto Rico, providing a 95% confidence level with a ±5% margin of error. The design with 12 age- and sex-matched healthy controls ensured robust comparative analysis, accounting for potential confounders [15]. In addition to matching for age and sex, exclusion criteria were applied to both groups to control for factors, such as recent infections or respiratory conditions that could bias results.

### 2.2 Participants

After receiving Institutional Review Board approval from Ponce Health Sciences University (Protocol #2305149716, approved 8/29/2023), participants were recruited through the Puerto Rico PCD Center, which is accredited by the PCD Foundation. Informed consent was obtained from all participants or their legal guardians, ensuring they were fully informed about the study's purpose, procedures, potential risks, and benefits. The study included 12 patients with PCD homozygous for the *RSPH4A* (c.921+3_921+6delAAGT) genetic variant and 12 age- and sex-matched healthy controls (HC). The PCD and HC groups were age-matched with an average age difference of ± 1.5 years.

For the PCD cohort, inclusion criteria consisted of patients aged five years and older with a confirmed diagnosis of PCD based on clinical phenotype and genetic testing for the *RSPH4A* variant. Healthy controls were selected based on the absence of known respiratory or immune disorders, non-smoking status, and no history of atopy or systemic inflammation. Exclusion criteria for the PCD cohort included the inability to perform the required exercise or the absence of genetic confirmation of PCD. For healthy controls, exclusion criteria included the presence of respiratory conditions (e.g., asthma), recent respiratory infections (<2 weeks), cardiovascular or neurological disorders, or a history of smoking.

### 2.3 Experimental Procedures

Baseline measurements were taken for all participants. Spirometry assessment of FEV1 was carried out using a calibrated spirometer (Platinum Elite Body Plethysmograph). nNO levels were measured using a validated chemiluminescence technique (CLD 88sp Chemiluminescence Nitric Oxide Analyzer, Dürnten, Switzerland), with participants exhaling through a mouthpiece with a resistor to ensure soft palate closure as per protocol [8]. SpO2 was monitored using a portable pulse oximeter (Nonin Model 7500).

Participants underwent a standardized six-minute exercise challenge test (ECT) on a stationary bicycle ergometer (ProForm 230 U Upright Exercise Bike) until reaching a target heart rate. The target heart rate (80% of the predicted maximum) was calculated using the standard formula (220 - age). After an initial 1-minute warm-up at low resistance, resistance was incrementally increased every minute until participants achieved the target heart rate. Once achieved, participants were required to maintain 80% of their predicted maximum heart rate for a full 1-minute interval. Heart rates were continuously monitored to ensure consistency. Participants were instructed to maintain a steady pedaling cadence (~60–70 RPM) throughout the test. The protocol was designed to stop early if participants reported severe fatigue, dizziness, or a drop in SpO_2_ below 88%, however, none of our participants required such measures.

Throughout the ECT, heart rate, blood pressure, and SpO2 were continuously monitored to ensure participant safety and gather real-time data on physiological responses. Post-exercise measurements were systematically repeated at multiple intervals. Spirometry was conducted at 5, 10, 15, and 20 minutes post-exercise to capture changes in lung function over time. nNO levels were measured immediately after the final spirometry at the 20-minute mark, while SpO2 was reassessed at 5 minutes post-exercise to evaluate the recovery of oxygen saturation following the exercise challenge. All measurements, including spirometry, nasal nitric oxide, and oxygen saturation, were conducted using standardized equipment and protocols to minimize measurement bias.

### 2.4 Data Collection and Analysis

Descriptive statistics were used to summarize the data, presenting medians and interquartile ranges or the study's primary outcomes, mainly nNO levels, FEV1, and SpO2. These variables were selected due to their clinical and physiological relevance in PCD. SpO_2_ was chosen to assess oxygenation changes, as exertional desaturation is a known issue in this patient population. FEV_1_, a standard measure of pulmonary function, allows for evaluating post-exercise changes in airway dynamics and safety. Nasal nitric oxide (nNO), a diagnostic biomarker for PCD, was included to investigate whether exercise impacts nitric oxide production, offering potential insights into disease-specific pathophysiology.

Comparative statistical analyses were conducted to assess the effects of exercise within and between the PCD and healthy control groups. For FEV1, a mixed-effects analysis or ANOVA was used to analyze the data. Paired t-tests were employed for nNO and SpO2 to compare pre- and post-exercise values within each cohort, offering insights into the immediate physiological changes induced by exercise. A significance level of p<0.05 was established for all statistical tests, ensuring that the findings were robust and meaningful. Subgroup analysis and interaction testing were not conducted, as the study focused on group-level comparisons between PCD patients and healthy controls. Missing data occurred for one healthy control participant who experienced exercise-induced bronchoconstriction and was excluded from further spirometry analysis. No imputation was applied, and analyses were performed with the available data. All statistical analyses were performed using GraphPad Prism version 10.2.3, a reliable tool for conducting both parametric and non-parametric tests and handling small sample sizes typically encountered in pilot studies.

## 3 RESULTS

### 3.1 Characteristics and Demographics of the Participants

In the study, 12 participants with PCD and 12 HC were recruited and underwent the ECT. The interquartile range (IQR) for age was 39.5 years for the PCD group and 38.5 years for the HC group. All recruited participants completed the study procedures, with the exception of one healthy control who experienced exercise-induced bronchoconstriction and was excluded from further spirometry analysis. Demographic characteristics, including age, sex, and relevant clinical history, were documented for all participants and are detailed in Table **1**.

### 3.2 Pulmonary Function

The immediate effects of aerobic exercise on pulmonary function were assessed by measuring FEV1. In the PCD group, pre-exercise FEV1 values averaged 53.3 L ± 10.2, and post-exercise values were 53.2 L ± 12.1. For the HC control group, pre-exercise FEV1 values were 94.4 L ± 12.2, and post-exercise values were 96.5 L ± 15.8. During the study, one participant in the HC group experienced exercise-induced bronchoconstriction (EIB), and further spirometry measurements were unavailable due to the administration of a rescue inhaler containing albuterol. Mixed-effects analysis or ANOVA was performed on the available pre- and post-exercise values and showed no significant changes in FEV1 for either the PCD (p=0.79) or the HC (p=0.22) groups Fig. (**1**).

Fig. (**1**) Line plot illustrating the Forced Expiratory Volume in one second (FEV1) values (%) in patients with Primary Ciliary Dyskinesia (PCD) and healthy controls (HC) before (Pre) and after (Post) a standardized six-minute exercise challenge test (ECT). The plot includes individual data points for each participant over time, with the occurrence of exercise-induced bronchoconstriction (EIB) indicated. No significant changes in FEV1 were observed post-exercise in either group (ns). Data are presented as individual values over time and created with BioRender.com.

### 3.3 Nasal Nitric Oxide (nNO)

nNO levels were measured to determine the impact of exercise on this parameter. The PCD group had a median baseline nNO level of 16.6 nL/min ± 15.1, which slightly decreased to 16.2 nL/min ± 14.6 post-exercise. In the HC group, baseline nNO levels were 302.9 nL/min ± 105.9, with a post-exercise level of 291.3 nL/min ± 119.6. Paired t-tests indicated no significant changes in nNO levels post-exercise in either group Fig. (**2**).

### 3.4 Oxygen Saturation

SpO2 levels were monitored to assess the immediate impact of exercise on oxygenation. In the PCD group, baseline SpO2 was 95.5% ± 2.1, increasing to 97.7% ± 1.5 post-exercise. In the HC group, SpO2 levels remained consistent, with baseline values at 97.9% ± 1.5 and post-exercise values at 98.9% ± 1.4. Paired t-tests showed a significant increase in SpO2 in the PCD group post-exercise (p<0.05), while no significant change was observed among the HC Fig. (**3**).

Fig. (**2**) Box plot illustrating the nasal Nitric Oxide (nNO) levels (nL/min) in patients with Primary Ciliary Dyskinesia (PCD) and healthy controls (HC) before (Pre) and after (Post) a standardized six-minute exercise challenge test (ECT). There were no significant changes in nNO levels post-exercise in either the PCD group or the HC group (ns). Data are presented as median with interquartile range, and individual data points are indicated. Created with Biorender.com.

Fig. (**3**) Box plot illustrating the oxygen saturation (SpO2) levels (%) in patients with Primary Ciliary Dyskinesia (PCD) and healthy controls (HC) before (Pre) and after (Post) a standardized six-minute exercise challenge test (ECT). The PCD group showed a significant increase in SpO2 post-exercise (**), while no significant change was observed in the HC group (ns). Data are presented as median with interquartile range, and individual data points are indicated. Created with Biorender.com

## 4 DISCUSSION

This study provides insights into the physiological responses of patients with PCD to aerobic exercise, an area underexplored in the literature. Understanding these responses is essential for developing effective management strategies that can enhance the quality of life for these patients. One of the primary findings of this study is the stability of pulmonary function, as evidenced by FEV1 levels, post-exercise in both cohorts. This result suggests that moderate aerobic exercise does not acutely impair pulmonary function in PCD as in other patients with EIB [16]. The stability of FEV1 in response to exercise aligns with findings in other chronic respiratory conditions, such as cystic fibrosis, where exercise regimens and pulmonary rehabilitation programs have been found to improve pulmonary symptoms and overall quality of life [14]. Previous studies on PCD have primarily focused on long-term pulmonary outcomes and have shown that regular physical activity can improve lung function over time [17]. However, these studies fail to report on the stability or instability of immediate pulmonary function. The findings presented in this study highlight the potential for exercise to be safely incorporated into PCD management protocols without the risk of immediate pulmonary function deterioration.

In addition to assessing pulmonary function through FEV1, the effects of exercise on NO levels remain understudied, with no existing literature specifically addressing this topic in the context of PCD. nNO is a critical diagnostic marker for PCD, with levels typically below 77 nL/min being indicative of the disease [18]. In our study, baseline nNO levels in the PCD cohort were significantly lower than the HC counterpart, aligning with this diagnostic criterion [9]. nNO is produced by NOS in the respiratory epithelium, and its low levels in the PCD disease process are thought to result from defects in this enzyme or its regulation [11]. The lack of change in nNO levels observed post-exercise in both PCD and HC suggests that moderate aerobic exercise does not directly influence the underlying physiological pathways responsible for reduced nNO production in PCD. This finding is relevant as it indicates that while exercise may have health benefits, it does not appear to modify the impaired Nitric Oxide synthesis characteristic of PCD.

Furthermore, this study reveals that SpO2 modestly improved post-exercise in the PCD cohort, a finding with important clinical implications that also warrants further investigation. Baseline SpO2 levels in PCD increased from 95% to 98% post-exercise, demonstrating a slight but significant enhancement in oxygenation following aerobic activity. This improvement contrasts with the stable SpO2 levels observed in the HC group, which remained consistent before and after exercise. The observed increase in SpO2 may be attributed to several physiological mechanisms that our study design was not able to assess. One possibility could be due to exercise’s known enhancements in pulmonary gas exchange *via* hyperventilation and increases in perfusion, which could potentially lead to increased alveolar recruitment and reduced areas of atelectasis [19].

Additionally, the bronchodilatory effects of exercise might help alleviate airway obstruction caused by mucus plugging, thereby improving overall oxygenation [20]. While these findings add to existing literature that report improved oxygenation and exercise tolerance in individuals with chronic respiratory diseases, it is important to note that the observed increase in oxygenation was modest and within the normal range [21]. However, the near-immediate improvement supports the notion that exercise can be beneficial for patients with PCD acting as a potential non-pharmacological intervention, enhancing physical fitness, respiratory efficiency, oxygen delivery, and overall quality of life.

Despite the valuable insights provided by this study, several limitations must be acknowledged. The small sample size of *n=*12 limits the generalizability of our findings, as it may not fully capture the variability in exercise responses among the broader PCD population. Additionally, our study exclusively included patients with the *RSPH4A* (c.921+3_921+6del) genetic variant. This genetic homogeneity restricts the applicability of our findings to patients with different genetic variants, who may exhibit varying physiological responses to exercise. Another significant limitation is the occurrence of EIB in one of the healthy control participants during the study. This observation suggests a potential high prevalence of undiagnosed EIB in Puerto Rico and highlights the importance of screening for this condition in studies involving physical activity.

Furthermore, our protocol did not include a cardiopulmonary exercise test (CPET), which could have provided additional insights into ventilatory and metabolic responses during exercise. The lack of end-tidal CO_2_ (ETCO_2_) monitoring prevents us from fully ruling out hyperventilation as a contributing factor to the observed rise in SpO_2_ post-exercise in the PCD group. Future studies incorporating CPET protocols with gas exchange measurements, including ETCO_2_, would help clarify whether hyperventilation plays a role in oxygenation changes observed during and after exercise in patients with PCD. The short-term nature of the study only allowed for the assessment of immediate post-exercise effects, necessitating long-term studies to understand the chronic impacts of regular aerobic exercise on pulmonary function, nNO levels, and oxygen saturation in patients with PCD. Lastly, the study did not explore the optimal intensity, frequency, or duration of exercise for maximizing health benefits.

## CONCLUSION

This study offers valuable insights into the immediate physiological effects of aerobic exercise in patients with PCD, suggesting its safety and potential benefits. The findings demonstrate that moderate aerobic exercise does not acutely impair pulmonary function, as indicated by stable FEV1 values. Additionally, the lack of significant changes in nNO levels suggests that exercise does not alter the mechanisms responsible for reduced nNO in PCD. Importantly, the significant improvement in SpO2 post-exercise sheds light on the potential of aerobic exercise to enhance oxygenation and respiratory efficiency in patients with PCD. These results support the integration of aerobic exercise into therapeutic regimens for PCD, promoting better clinical outcomes and quality of life. However, the limitations of the study, including a small sample size and genetic homogeneity, draw attention to the need for further research with larger, more diverse cohorts to fully understand the long-term benefits and optimal exercise protocols.

## Figures and Tables

**Fig. (1):**
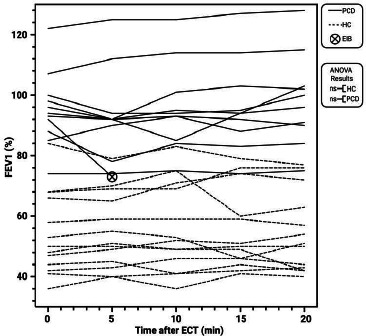
FEV1 response to aerobic exercise.

**Fig. (2):**
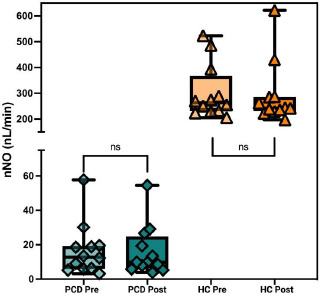
Nasal Nitric Oxide response to Aerobic Exercise.

**Fig. (3):**
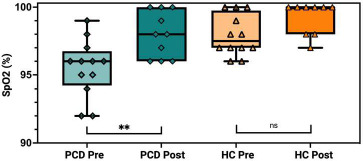
Oxygen saturation response to Aerobic Exercise.

**Table 1:** Demographic and clinical characteristics.

**Characteristics**	**Percentage (%)**	
	PCD Group	HC Group
**Gender (F)**	50	50
**Age (>21)**	58	58
***RSPH4A* Homozygosity**	100	0
**nNO below 77nL/min**	100	0
**Exercise Induced Bronchoconstriction**	0	8

**Abbreviations:** PCD: Primary Ciliary Dyskinesia; HC: Healthy Control; nNO: Nasal Nitric Oxide

## Data Availability

The data supporting the findings of the article will be available from the corresponding author [G.G-D] upon reasonable request.

## LIST OF ABBREVIATIONS

PCD: = Primary Ciliary Dyskinesia
FEV1: = Forced Expiratory Volume in 1 second
SpO2: = Oxygen Saturation
nNO: = Nasal Nitric Oxide
HC: = Healthy Control
ECT: = Exercise Challenge Test
NOS: = Nitric Oxide Synthase
EIB: = Exercise Induced Bronchoconstriction
IQR: = Interquartile Range

## STANDARDS OF REPORTING

STROBE guidelines were followed.
